# siRNA Genome Screening Approaches to Therapeutic Drug Repositioning

**DOI:** 10.3390/ph6020124

**Published:** 2013-01-28

**Authors:** Olivia Perwitasari, Abhijeet Bakre, S. Mark Tompkins, Ralph A. Tripp

**Affiliations:** Department of Infectious Diseases, University of Georgia, Animal Health Research Center, 111 Carlton Street, Athens GA 30602, USA; E-Mails: olperwit@uga.edu (O.P.); bakre@uga.edu (A.B.); smt@uga.edu (S.M.T.)

**Keywords:** RNAi, siRNA, miRNA, genome, antiviral, HTS, therapeutic, pathway, virus, bacteria, pathogen, reposition, repurpose, host genes

## Abstract

Bridging high-throughput screening (HTS) with RNA interference (RNAi) has allowed for rapid discovery of the molecular basis of many diseases, and identification of potential pathways for developing safe and effective treatments. These features have identified new host gene targets for existing drugs paving the pathway for therapeutic drug repositioning. Using RNAi to discover and help validate new drug targets has also provided a means to filter and prioritize promising therapeutics. This review summarizes these approaches across a spectrum of methods and targets in the host response to pathogens. Particular attention is given to the utility of drug repurposing utilizing the promiscuous nature of some drugs that affect multiple molecules or pathways, and how these biological pathways can be targeted to regulate disease outcome.

## 1. Introduction

The classical pathway of drug development generally involves two approaches. The first is large-scale screens of chemical libraries, measured in the hundreds of thousands of compounds, against identified disease targets with a measurable endpoint of activity [1]. Alternatively, when precise target structures are known, structure-based virtual screening is used to select a more limited set of compounds for biological screening [2]. From these efforts, approximately 324 unique drug targets, including 266 human and 58 pathogen targets, have been identified [3]. Unfortunately, less than 6% of drugs licensed between 1988 and 2000 acted on novel molecules or domains [3], indicating a bottleneck in the development of new therapeutics. 

While efforts continue to develop new drugs targeting new molecular entities, there is increasing interest in using existing drugs for new indications, *i.e.* drug repurposing or repositioning [4]. These compounds not only have established safety and pharmacokinetic profiles, but they have also been through bulk manufacturing and formulation development making repositioned drug development faster and less risky than traditional pipelines [4]. Moreover, the use of licensed drugs in combination to achieve a novel pharmacologic effect provides yet another approach for drug repurposing [5]. With the increased emphasis on use of existing cellular molecules, new screening approaches are moving away from compound identification and towards target identification. 

### 1.1. RNAi Dependent Gene Silencing Pathways

**Figure 1:**
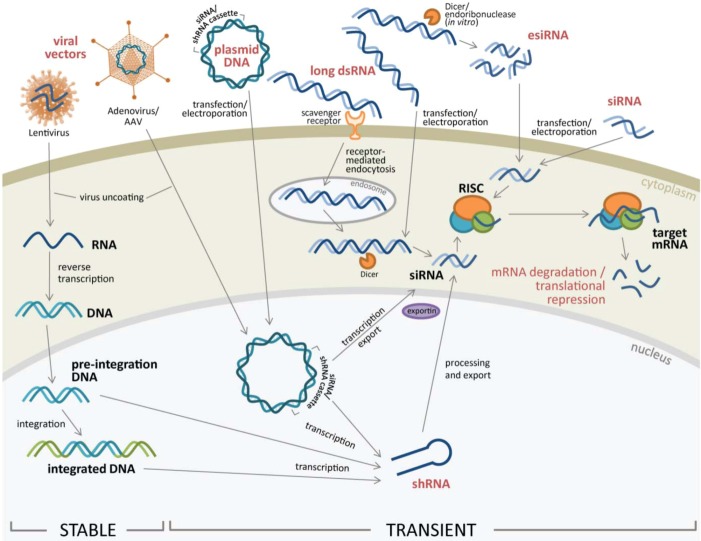
Types of RNAi constructs introduced into target cells for silencing. Naked RNAi constructs can be introduced to target cells in forms of siRNA (small interfering RNA), esiRNA (endoribonuclease-prepared siRNA), or long dsRNA using variety of transfection protocols such as lipid-based transfection reagents or electroporation, or active uptake by target cells through receptor-mediated endocytosis. Once inside the cytoplasm, RNAi constructs can be further processed by host endonucleases or directly loaded into RISC (RNA-induced silencing complex) to facilitate gene expression knock down. Alternatively, for hard-to-transfect cells, RNAi can be introduced using viral vectors, such as lentivirus or adenovirus/adeno-associated virus (AAV) vectors carrying siRNA or shRNA expression cassettes. Once transcribed, shRNA can be further processed and exported out to cell cytoplasm for silencing gene expression.

RNA interference (RNAi) has evolved over the past fifteen years from a Nobel prize-winning discovery to an established mainstream research tool for discovery and for treatment of some diseases. First described in *Caenorhabditis elegans*, it was shown that introduction of exogenous, double-stranded RNA blocked protein production through sequence-specific degradation of messenger RNA (mRNA) [6]. Subsequently, RNAi was shown to be mediated by 21-23 base pair (bp) small-interfering RNA (siRNA) derived from dsRNA by a family of RNase III-like enzymes, including Dicer (reviewed in [7]). As illustrated in Figure 1, siRNA duplex(es) introduced into the cell enter the RNA-induced Silencing Complex (RISC) pathway where conserved components including the Argonaute (Ago) proteins and other accessory factors unwind and cleave the passenger strand in the siRNA duplex leaving the guide strand to pair with the target mRNA via perfect sequence complementarity. The guide strand provides specificity for the activated RISC, leading to cleavage of the target mRNA abolishing protein production. Non-coding RNAs (ncRNA) pre-date the discovery of RNAi. Typically, ncRNAs have been classified by size into long non-coding RNAs (lncRNAs) or small non-coding RNAs, and comprise 26 or more functional categories reflecting the diversity of ncRNA function [8]. siRNA and microRNA (miRNA) are similar as both use RISC pathway components but differ in their mode of gene silencing and outcome. siRNAs typically exhibit full length complementarity with target mRNAs in the coding region causing gene-specific knockdown via target mRNA cleavage while miRNAs show a 6-8 nt complementarity with target gene 3'-UTR via a miRNA “seed region” and block translation by interfering with the initiation or elongation phase of protein synthesis. In 1993 the first putative small regulatory ncRNA, a 22 nucleotide (nt) *lin-4* transcript, was shown to have complementarity to *lin-14* mRNA [9]. Previous work demonstrated the ability of *lin-4* to negatively regulate LIN-14 protein expression, but the mechanism of regulation was undefined [10]. It was not until 2000 that a second ncRNA was described, *i.e. let-7*, a 21 nt post-transcriptional negative regulator expressed temporally during *C. elegans* development [11]. Though initially only a few ncRNAs were described in *C. elegans*, *Drosophila*, and humans [10] present numbers extend to several thousand in these model species [8]. 

Unlike siRNAs, miRNAs are genome-encoded and transcribed individually, in clusters, or within an intron of a protein-coding gene [7]. The miRNA is transcribed by RNA polymerase II as a capped and polyadenylated primary miRNA (pri-miRNA). The pri-miRNA folds into a stem-loop structure that is cleaved from the remaining transcript in the nucleus, forming a pre-miRNA. In mammals, the pre-miRNA is exported from the nucleus and processed by Dicer family nucleases, which then enters the RISC pathway in a similar fashion as siRNA. The miRNA unwinds and associates with the RISC and directs the RISC to complementary miRNA recognition element (MRE) sequences generally found in the 3’ UTR of the mRNA. miRNAs having complete complementarity to the mRNA will direct cleavage and degradation of the mRNA by RISC, while miRNAs having partial complementarity (mediated by a 6-8 nt seed region) drive translational inhibition of the transcript [7]. An explosion of research in the miRNA field has shown miRNAs to govern protein expression and affect a wide range of biological activities including stem cell development/differentiation, immune cell differentiation and activation, and oncogenesis [12]. Moreover, miRNAs have been shown to be involved in host-pathogen interactions whether encoded by the host cell or the invading pathogen.

### 1.2. Using Human Genome Data to Generate siGenome Libraries

Since the human genome sequence was published in 2001 [13], a time coinciding with early elucidation of the RNAi pathway, efforts to develop siRNAs targeting the entire genome have been pursued. Due to an incomplete understanding of the sequence and structural features that drive siRNA and miRNA based silencing, initial reagents suffered from unwanted off target effects [14] which have been subsequently reduced given better insights into siRNA and miRNA structure and function [15]. A common concern with siRNA design is the potential overlap with miRNA activity via seed site identity [14,16,17], a feature that cannot be completely avoided. Prevalent computational algorithms rely on determining secondary structure based matches between miRNA seed sites and MREs and ascribe scores to each pair to identify putatively strong regulators which are then extended by using either inter-species conservation matrices or other structural features to yield a large collection of miRNA target prediction algorithms [18]. Novel alternate approaches to identify true miRNA targets are based on isolating miRNA:target transcript pairs from RISC complexes using techniques as HITS-CLIP [19] or PAR-CLIP [20] and next generation sequencing tools. These tools complement computational approaches to identify true siRNA/miRNA pairing rules and add to our understanding of the RISC pathway. With this realization, algorithms defining candidate miRNAs and miRNA targets along with experimentally defined miRNAs and targets sites are being used to design candidate siRNAs that have low non-specific or off-targeting potential. A secondary concern is the non-physiological concentrations of siRNA/miRNA used in RNAi screens. Commercial siRNAs/ miRNAs are typically chemically modified to extend their intracellular stability and longevity, and thus can potentially trigger secondary signaling cascades contributing to off-target phenotypes. While the genomes of simpler model organisms, such as *C. elegans* and Drosophila are amenable to partial or full-genome RNAi screens, the delivery of siRNAs in mammalian systems is a greater challenge. In 2003, HeLa cells were screened with a library of 510 commercially synthesized siRNAs to identify genes involved in TRAIL-induced apoptosis [21]. Subsequently in 2004, two groups published large scale screens using human cells and different approaches. One generated a library of retroviral vectors encoding >20,000 shRNAs targeting almost 8,000 human genes [22], while another generated both mouse and human short hairpin RNA (shRNA) libraries targeting 9,610 human and 5,563 mouse genes, respectively [23]. Later that year, siRNAs from a cDNA collection of approximately 15,000 human genes were made using >5,000 recombinant RNase III endoribonuclease-prepared siRNAs (esiRNAs) and used to screen HeLa cells [24]. At that time, full-genome siRNA libraries (siGenome) were being developed; however beyond siRNA design, the complexity of efficient host cell delivery and silencing using a high-throughput screening (HTS) format made siGenome screening a significant challenge (reviewed in [25]). Genome libraries of siRNAs became commercially available in 2005, and the first genome screen using a siRNA library produced by Dharmacon was published in 2007 [26,27]. 

### 1.3. Considerations for RNAi Screens

The commercial availability of siRNA libraries covering the human genome has provided the impetus for widespread genomic RNAi screening across biologic disciplines. More than 70 genome scale screens in model organisms and mammalian systems have subsequently been published, identifying new targets against infectious disease, novel oncogenes, sensitizers to existing drugs, as well as more basic discoveries [28]. Beyond specific discoveries, these screens provide numerous insights into improved methods for genome-scale screening. While RNAi screening on this scale may not yet be considered commonplace, guidelines for design, execution, and analysis of these screens have been established and depending on the model system, a genome-wide screen can be considered routine. 

There have been a number of publications reviewing approaches for genome-scale RNAi screens [27,29]; however, key points remain to be considered. The core objective behind RNAi screens is to identify class(es) of genes that have an impact on the biological question under study and extend those findings to elucidate gene function in pathways and biological networks. Thus, the first decision in undertaking an RNAi screen is to determine the biological question to address as this dictates the choice between a genome-wide RNAi screen and a targeted sub-library approach. Often the choice between the two is dictated by the research budget available, time-points to be assayed, and feasibility of endpoint assays. Frequently, pilot screens with targeted libraries are employed to generate preliminary hypotheses for follow up to elucidate pathways involved. Focused library screens are often more cost-efficient and an appropriate option when preliminary findings points toward specific class of cellular molecules, but may miss important secondary regulators important for the phenotype. Of particular interest, drug target siRNA libraries can be valuable for discoveries of new drug targets. These libraries were compiled based on druggability properties of proteins where their folding favors interaction with drug compounds thus increasing the chance of pharmacological inhibitors availability [30]. 

The model system chosen for study typically dictates which endpoints can be used and this strongly influences the siRNA approach. For example, models with cell death as a selectable marker can use a shRNA based approach to identify surviving cells as hits. In contrast, more subtle phenotypes might require a siRNA screening approach. The type of screen employed is also determined by instrumentation and technology available. For high throughput screens, the endpoint must be amenable to rapid high throughput measurement since genome-wide screening in replicates often generates tens of thousands of samples for analysis. The number of samples also requires the endpoint to be stable enough for consistent measurement over time. Using reporter systems as endpoints have been a popular approach for these reasons. More recently, high-content image analysis technologies have made significant improvements, enabling high-throughput and high-content image analysis of reporter systems or immunofluorescent staining to allow for tiered analysis of protein co-localization or other sub-cellular features as endpoints [31]. Importantly, the endpoint assays used must be cost-effective. An assay that is affordable at the single sample level can be prohibitively expensive when >10,000 measurements are required, or too variable, or cumbersome for a HTS RNAi genome-scale screen.

### 1.4. miRNA Screens

The understanding of the importance of miRNA gene regulation has grown, and as genome-scale screens have identified genes, the role of miRNAs regulating their expression is of interest. MicroRNAs are 20-24 nt RNAs derived from genome-encoded stem-loop structures that target mRNA for degradation or inhibit translation. MicroRNAs primarily base pair with a 6-8 nt “seed region” for specificity, and given this relatively small sequence complementarity, an individual miRNA may potentially target numerous mRNA targets [32]. The human genome is now annotated with >2,000 mature miRNAs (miRBase v19) [33] and the majority of mammalian genes are predicted to be regulated by miRNAs [34] adding to the complexity of the analysis.

Tools are now available to study the function of miRNAs through inhibition of miRNA activity or addition of exogenous mature miRNAs. While siRNA screens should uncover role of individual genes in the cellular processes of interest, miRNA screens allow researchers to discover interconnected genes and pathways regulated by single miRNA. Paired analysis of miRNA mimics and hairpin inhibitors enables gain/loss of function studies for a given miRNA. While hundreds of potential miRNAs have been identified, the smaller scale of screening compared to genome-wide screening presents new opportunities to identify genes and regulatory mechanisms for a given biological process. While identification of an individual miRNA may not define an individual mRNA as relevant, bioinformatics tools identifying miRNA targets can provide candidates genes for targeted siRNA screening. Where siRNA screen data is available, miRNA mimic/inhibitor results can be correlated with siRNA screen gene targets to not only identify high-confidence hits, but also provide potential regulatory mechanisms and/or therapeutic targets.

## 2. RNAi Delivery to Cells

RNAi involves introducing nucleic acids into host cells to modulate expression of a targeted gene of interest [35,36]. This technology utilizes the host cell RNAi machinery which is widely conserved in most eukaryotes [6,37]. In synthetic RNA silencing, RNAi delivery is in a “black box” because efficient delivery in some cells types, and *in vivo*, has yet to be fully realized; however for many common cell types, exogenous siRNAs and shRNAs can be introduced using a variety of methods [36,38]. Types of RNAi and different methods for introducing RNAi into cells are reviewed in this section.

### 2.1. RNAi Delivery Methods into Target Cells

Efficient delivery of siRNA/shRNA into target cells is crucial to achieve maximal gene repression. As reviewed below and summarized in Table 1, there are multiple factors that influence the choice of RNAi delivery method *in vitro* including cell types to be targeted, transfection reagent, and whether stable or transient silencing is desired. 

**Table 1:** RNAi delivery systems for different cell types.

	**Transient RNA interference**			**Stable RNA interference**	
**Cell type**	Lipid-based transfection	Electroporation	Adenovirus/AAV vectors	Lentivirus vectors	Retrovirus vectors
Most secondary and transformed cell lines (adherent or suspension)	X	X	X	X	X
Difficult to transfect cells		X	X	X	X
Primary non-transformed cells (dividing)		X	X	X	X
Primary non-transformed cells (non-dividing)		(nucleofection)	X	X	
Growth-arrested and contact-inhibited cells				X	

#### 2.1.1. Lipid-Based Transfection and Electroporation of Nucleic Acids

Most of the secondary or transformed cell lines used in the laboratory RNAi studies are amenable to RNAi delivery and include HEK293, A549, Vero, and HeLa cells. This is in part because these cells allow convenient introduction of siRNA molecules or plasmid DNA containing shRNA or siRNA expression cassettes using widely available lipid-based transfection reagents [39,40,41]. This method works through the formation of cationic lipid-nucleic acid complexes, which are taken up by target cells through endocytosis. Many lipid-based transfection reagents are also pre-optimized for common cell lines, allowing researchers to achieve maximal transfection efficiency. 

Another less common method to introduce siRNA or expression plasmids into difficult to transfect (e.g. Jurkat and THP-1 cells) and primary cells is electroporation [39,41]. This procedure utilizes electrical pulses which transiently allow for cellular membrane permeabilization and entry of nucleic acid molecules. Unlike traditional transfection and electroporation methods that rely on cell division for nuclear entry of nucleic acid, specific electroporation methods such as nucleofection allow for entry of nucleic acids into nuclei of non-dividing cells, such as neurons and other non-dividing primary cells. However, electroporation often results in a higher level of cell death compared to lipid-based methods. 

#### 2.1.2. Viral Vectors to Transfer RNAi into Target Cells

Viral vector delivery methods take advantage of the ability of a virus to infect and transfer nucleic acid into target cells. Several viral vectors are commonly used for RNAi silencing, including adenovirus/adeno-associated virus (AAV), retrovirus, and lentivirus [42,43,44]. Generally, viral vectors are designed to be replication incompetent, thus RNAi expression cassettes in the form of a circular dsDNA (for adenovirus/AAV vectors) or ssRNA (for retrovirus/lentivirus vectors) need to be packaged in specialized cell lines expressing virion proteins [44]. While lentivirus, adenovirus, and AAV vectors are able to infect broad ranges of dividing and non-dividing cells, retrovirus vectors only efficiently infect dividing cells [42,45,46,47]. Both lentivirus and retrovirus vectors result in lower transduction efficiency but allow for stable integration of shRNA expression cassette into host cells’ genome which results in long-term knock down of genes of interest. Adenovirus/AAV vectors, on the other hand, achieve near 100% transduction efficiency although rarely result in chromosomal integration; therefore, it is more suitable for a transient knock down of gene expression [48,49].

### 2.2. Different Types of RNAi Introduced for RNA Silencing

#### 2.2.1. siRNA and esiRNA

siRNA duplexes with phosphorylated 5'-ends and hydroxylated 3'-ends with two overhanging nucleotides of approximately 21-base pairs in length can be synthesized chemically or by *in vitro* transcription [37]. As noted above and illustrated in Figure 1, siRNA duplexes can be introduced into target cells using lipid-based transfection or electroporation [39,41,50]. As with endogenous cellular miRNAs, once inside the cytoplasm siRNA molecules are loaded into RISC where siRNA guide strands bind to complementary sequence on target mRNA and induce cleavage of target mRNA by endonuclease Ago2 within the RISC complex [51,52]. Alternatively, imperfect base-pairing of siRNA to complementary mRNA induces translational silencing of target mRNA [53]. In addition to synthetically generated siRNAs, pools of siRNA molecules targeting a specific gene, termed esiRNAs, can be generated by cleaving long dsRNA *in vitro* by Dicer or RNase III [54]. As esiRNA consists of pools of siRNA oligos targeting a single mRNA, this method often results in more specific protein expression knock down while minimizing off target effects.

There are several disadvantages associated with this siRNA and esiRNA methods of gene silencing, including the transient nature of protein expression silencing, inability of several cell types to be transfected or electroporated, stability of RNA molecules, and activation of the host cell innate immune response. Innate immune responses can be triggered by introduction of foreign RNA and recognition by membrane-associated and intracellular pattern recognition receptors (PRRs) that include Toll-like receptors (TLRs) and Retinoic-acid inducible gene (RIG-I)-like receptors (RLRs), although this is less common compared to longer dsRNA molecules (>25 base pairs) [55,56,57]. Several modifications of siRNA molecules, such as phosphorothioate modification to RNA backbone, and 2’-O-Methyl or 2’-Fluoro modification to nucleotides, have been reported to help evade host innate immune detection while increasing the stability of RNA molecules [58,59,60].

#### 2.2.2. Long dsRNA

Similar to siRNA duplexes, long dsRNA molecules containing siRNA sequences can be introduced directly into target cells by transfection and electroporation, as illustrated in Figure 1. Additionally, long dsRNA can also be taken up by cells through receptor-mediated endocytosis. Several plasma membrane-associated receptors that can bind to RNA molecules, such as scavenger receptors (SR-B1) and PRRs (TLR3) have been identified to mediate this process [61,62]. Once inside the cytoplasm, these dsRNA molecules can be processed by Dicer to generate siRNA duplexes via RISC and induce silencing of target mRNA. Since dsRNA molecules are recognized by membrane-associated and intracellular PRRs, innate interferon response is triggered upon their presence, thus being a disadvantage as an RNAi tool [37,57]. 

#### 2.2.3. Expression Vectors Containing RNAi Cassettes

Unlike dsRNA, circular dsDNA containing siRNA or shRNA expression cassettes can be introduced into target cells with minimal triggering of innate immune responses. Like siRNA and long dsRNA, circular expression plasmid DNA can be transfected or electroporated into cells as illustrated in Figure 1 [50,63]. Additionally, circular dsDNA can also be transduced into target cells using viral vectors, such as adenovirus/AAV vectors [49]. Circular DNA containing shRNA or siRNA expression cassettes downstream of RNA polymerase III promoters must enter the nucleus to access cellular transcription machinery for expression of the shRNA or siRNA. Thus, only nucleofection and viral vectors will allow circular dsDNA plasmid access into the nucleus of non-dividing cells, as previously described. Once shRNA or siRNA molecules are transcribed, they are exported out to cytoplasm using cellular exportins. In the cytoplasm, shRNA can be further processed by Dicer to yield short siRNA duplexes that can be loaded into RISC for gene silencing.

#### 2.2.4. Stable Gene Expression Silencing using Lentivirus or Retrovirus Vectors

Most siRNA molecules lack the ability to self-replicate, thus result in transient knock-down of the gene of interest. Transient gene knock-down generally lasts for a day or two following transfection or transduction as RNAi is eventually lost due to dilution by cellular division [64,65]. Unlike transient RNAi silencing, once target cells are infected or transduced using lentivirus or retrovirus vectors, viral reverse transcriptase can begin to reverse-transcribed ssRNA containing shRNA sequences as both are packaged inside the viral vector [43]. Subsequently, viral integrase catalyzes stable integration of DNA package into the cell chromosome which can then be replicated through cellular divisions (Fig. 1), thus shRNAs are stably expressed throughout cellular passage [66]. Although both lentivirus and retrovirus vectors often result in less than 30% transduction efficiency, selection markers such as puromycin resistance are often included to allow selection for cells with stably-integrated shRNA expression cassettes in presence of the appropriate selection antibiotic. Additionally, if non-constitutive knockdown of gene expression is desired, inducible promoters can be used to direct expression of shRNA [67]. Although both lentivirus and retrovirus vectors result can result in stable gene expression knock down, unlike lentivirus vectors, retroviral vectors such as murine leukemia virus (MuLV) vectors are unsuitable for transduction of non-dividing cells as they can only stably integrate into genomes of actively dividing cells [42,45,46,47]. 

## 3. Unraveling the Biological Implications of Pathogens using HTS Screening

A robust approach is needed to unravel the biology of eukaryotic cellular responses to environmental, developmental, and pathogenic challenges. This should involve combining approaches that include whole genome transcriptome studies, proteomics, metabolomics, epigenetic modifications and tying the findings of these approaches using systems biology tools to identify the foundations perturbed. RNAi screens have been employed in a plethora of settings to identify pertinent biological networks. These studies not only identify genes that have important roles in these networks, but also discover unknown modes of cell regulation and control. As described below, this feature has allowed for identification of new targets for drug repurposing so that existing drugs can be used for novel interventions and therapies. Since eukaryotic gene regulatory networks tend to be highly conserved, this approach also aids in identifying the evolution of stress-response pathways. While this review focuses on the findings of RNAi screens from both intrinsic and extrinsic stress-response pathways, it must be remembered that RNAi knockdown is not equivalent to knockout, and that host gene networks identified may not be similar in normal homeostasis *versus* when under duress, and that silencing of a response in one pathway can be overcome by use of alternate or redundant pathways.

### 3.1. RNAi Screens for Human Bacterial Pathogens

RNAi has been used successfully to identify host factors that are required for replication or involved in pathogenesis of several important bacterial, fungal, protozoal, and viral pathogens and is summarized in Table 2. These studies highlight the impact of RNAi screening approaches in identifying known and novel cellular networks used by pathogens to survive in the host. While this summary focuses on intracellular pathogens, extracellular pathogens may also modulate these pathways to escape immune surveillance. Recently, 21,300 siRNAs were evaluated in Drosophila S2 cells to identified 305 host genes that modulated *Listeria monocytogenes* infection and replication, and many of the genes identified constituted important choke points in multiple cellular pathways such as protein biosynthesis, ribosome components, proteasome mediated degradation, and cytoskeleton reorganization [68]. 

A recent study has also identified seven host cell kinases (ACVRL1, CDK5R1, CSNK1A1, CSNK2B, PDGFRB, SNARK, and TTK) whose depletion blocked *L. monocytogenes* spread in human cells [69]. Interestingly, less than 13% of the genes identified [68] had adverse effects on cell viability, and importantly genes from vesicular trafficking and cytoskeleton component reorganization pathways were also found to regulate replication of another intracellular pathogen, *Mycobacterium fortuitum*. A similar screen of >21,000 siRNAs in the same system was used to identify host factors important for replication of mycobacteria, and accordingly several conserved genes were identified between mycobacteria and other human bacterial pathogens such as *Staphylococcus aureus* and *Escherichia coli* [70]. The initial screen identified 86 siRNAs that decreased infection and targeted genes involved in lipid metabolism, chromatin structure and organization as well as proton transport, vesicle trafficking, actin cytoskeletal organization, and signal transduction. Scavenge receptors, specifically CD36, was found to be essential for *M. fortuitum* infection but not phagocytosis [70]. Another gene CG7228 (renamed *Peste*) was found to be important for uptake of *M. smegmatis*, *M. fortuitum,* and *L. monocytogenes*. *Peste* reconstitution in HEK293 cells (which are normally refractory to *M. fortuitum* infection) allowed *M. fortuitum* infection and promoted *S. aureus* and *E. coli* infection. In a related study [71], 1000 genes were analyzed by RNAi revealed lysosomal enzyme β-hexosaminidase to be essential for controlling mycobacterial infection. A more comprehensive study using RNAi knockdown of host kinases and phosphatases involved in *M. tuberculosis* infection identified 41 genes that regulate *M. tuberculosis* replication [72]. Of these 41 genes, 11 genes led to a significant knockdown of the lab H37Rv strain as well as two clinical multiple drug resistant isolates validating the roles of these genes in mycobacterial replication. This was later extended to a genome-wide screen using ~18,000 siRNAs targeting the human transcriptome to identify genes essential for replication of multiple lab and clinical strains of *M. tuberculosis*. 

**Table 2:** Summary of RNAi screens to identify host factors involved in pathogenesis.

Pathogen	Screen size	Gene family target	Validated candidates	Major pathways identified	Ref.
***Bacterial***					
***L. monocytogenes***	~21,300	Whole genome	305	Protein biosynthesis, proteasomal degradation, cytoskeletal networks	[68]
	779	Kinome	7	Kinase networks	[69]
***M. fortuitum***	~21,300	Whole genome	2	Vesicular transport and cytoskeletal networks	[68]
***M. fortuitum and other species***	~21,000	Whole genome	86	Lipid metabolism, chromatin organization, proton transport, vesicular transport, actin cytoskeleton, and signal transduction	[70]
***M. marinum, M. tuberculosis***	1,000	n.a	1	β-hexosaminidase	[71]
***M. tuberculosis H37Rv***	744 + 288	Kinases + phosphatases	41	Signaling networks	[72]
***M. tuberculosis***	18,174	Whole genome	275	Multiple pathways	[73]
***C. caviae***	16,128	Whole genome	54	Multiple pathways	[74]
	7,216	Partial genome	226	Kinases Abl and PDGFR	[75]
***P. aeruginosa***	80	Actin cytoskeleton associated genes	4	Abl kinase, Crk adaptor protein, Rac1 small GTPase, Cdc42, and p21 kinase components	[78]
***S. typhimurium***	6,978	SopE-associated host proteins	72	COPI complex, lipid biosynthesis	[79]
	~22,000	Whole genome	252	Cellular development, cellular growth, carbohydrate metabolism	[80]
***Brucella spp.***	240	ER associated proteins	52	Inositol metabolism, eukaryotic unfolded protein response (UPR)	[81]
***F. tularensis***	~47,400	Whole genome	~200	Multiple pathways	[82]
***Fungal***					
***C. albicans***	7,216	Genes shared among metazoans	184	Multiple pathways	[83]
***C. neoformans***	410	Targeted subset of multiple pathways	57	Multiple pathways	[84]
***Protozoal***					
***P. falciparum***	727	Kinome	5	Signaling networks	[85]
	n.a.	Gene specific	1	Scavenger receptor B1	[86]
***Plasmodium spp.***		Gene specific	3	oxr1, argK & prs1	[87]
***T. cruzi***	21,127	Whole genome and gene specific studies	162	Multiple pathways	[88,89,90,91]
***Viral***					
***Drosophila C virus***	21,000	Whole genome	66	Ribosomal proteins, translation	[116]
***Human Immunodeficiency virus (HIV)***	n.a.	Gene specific	1	Human Spt5 transcription elongation factor	[94]
	n.a.	Gene specific	1	DBR1 splicing factor	[95]
	5,000	Druggable gene targets	4	Multiple pathways; kinases	[96]
	21,121	Genome wide	273	Multiple pathways	[97]
	19,709	Genome wide	311	Multiple pathways	[98]
***Human Immunodeficiency virus (HIV)***	622 + 180	Human kinase + phosphatase shRNAs	14	Multiple pathways	[99]
	30	Targeted genes	15	Kinases, vesicular transport, and others	[100]
	232	DNA repair pathway	35	Base excision pathway repair	[101]
	19,121	Whole genome	114	PAF1 complex	[102]
	12	Autophagy pathway targeted shRNAs	5	Autophagy pathway components	[103]
***Influenza virus***	13,071	Drosophila whole genome	~100	Multiple pathways	[104]
	17,877	Human whole genome siRNA library	120	Multiple pathways	[105]
	1,745	Targeted influenza protein interactors	616	Multiple pathways	[106]
	22,843	Human whole genome	287	Multiple pathways	[107]
	19,628	Human whole genome	295	Multiple pathways	[108]
	481	Human protease siRNA library	5	c-AMP , NF-κb, and apoptosis	[109]
***Hepatitis C virus (HCV)***	~4,000	Druggable targets	9	Multiple pathways	[117]
	62	HCV–host interactions	26	Multiple pathways including Dicer	[118]
	510	Human kinase library	3	Csk, Jak1, and Vrk1	[119]
	140	Membrane trafficking family	16	Clathrin coated pit proteins, actin polymerization, AP2 adaptor, ubiquitin ligase, ER/Golgi trafficking	[110]
	140	Membrane trafficking family	7	Endosomal trafficking, lipid organization, and actin polymerization	[120]
	21,094	Human whole genome	96	Multiple pathways	[121]
***Vesicular stomatitis virus (VSV), lymphocytic choriomeningitis (LCMV), and parainfluenza virus (PIV) 5***	22,909	Human whole genome	72	Coatomer complex 1 and other pathways	[111]
***Ebola virus***	720	Kinases and phosphorylases	~190	Multiple pathways	[112]
***Vaccinia virus***	7,000	Drosophila druggable genes library	188	Multiple pathways	[114]
	440	Kinases + phosphatases + regulator factors	7	AMPK kinase, endocytosis	[113]
***Human papillomavirus (HPV)***	21,121	Human whole genome library	96	DNA demethylation, histone acetyl transferases	[122]
***Coxsackie and polio virus***	5,492	Human druggable genome library	117	Rab GTPases, Src tyrosine kinases, and phosphatase networks	[123]
***West Nile virus (WNV)***	21,121	Human whole genome library	305	Multiple pathways	[124]
***Dengue Virus***	22,632	Drosophila whole genome	116	Multiple pathways	[125]
	119	membrane trafficking genes	6	Clathrin-mediated endocytosis	[126]

n.a.= not applicable / not available

Of the siRNAs tested, 1,138 genes were found to modulate *M. tuberculosis* replication ±1.5 fold relative to mock transfected cells, and 509 were validated by an independent secondary screen of which 275 genes regulated the replication of a number of clinical isolates [73]. Similar screening for *Chlamydia caviae* identified 54 genes important for replication, and several overlapped as significant for *L. monocytogenes* screens described previously [74]. Parallel studies by other groups have identified Abl kinase and the PDGFR signaling pathway [75], and the MEK-ERK signaling pathway as important for *Chlamydia* infection [76]. Similarly, RNAi screening helped identify determinants of chemotaxis in *Pseudomonas aeruginosa* infection [77,78], and identified 72 host factors that affect SopE-mediated *Salmonella typhimurium* entry [79]. A similar approach identified 252 host genes that modulate *S. typhimurium* replication, and of these, 39 genes when down-regulated increased bacterial replication representing newly discovered anti-bacterial defenses [80]. The zoonotic *Brucella* spp. pathogens replicate in intracellular compartments that contain components of the ER pathway and a recent study identified 52 host factors that when depleted either up regulated or induced *Brucella* replication [81]. A recent RNAi based study also reported identification of genes that regulate replication of *Francisella tularensis* [82]. Thus, numerous siRNA screens have been employed to identify genes at the host-bacteria interface. A number of drugs have been repurposed as novel anti-bacterial treatment recently and include molecules against *M. tuberculosis* and *P. aeruginosa* (Table 3).

**Table 3:** List of repositioned drugs currently in different stages of clinical trial.

Drug	Original indication	New indication	Clinical trial stage	Ref.
***Infectious Diseases***				
**Anti-bacterial**				
PNU-100480	MRSA	Tuberculosis	Phase I clinical trail	[145]
Sulphamethaxazole + Trimethoprim	Generic antibacterial	Tuberculosis	Clinical use	[146]
Raloxifen	Osteoporosis + breast cancer	*P. aeruginosa*	Preclinical	[147]
**Anti-protozoal**				
Astemizole	Antihistamine	Malaria	preclinical	[148]
Dapsone	Leprosy	Malaria	phase 3 completed	[117]
Amphotericin	Antifungal	Leishmaniasis	phase 3 completed	[117]
DB289	Pneumocystis	Malaria and African trypanosomiasis	phase 2 completed	[117]
Eflornithine	Cancer	African trypanosomiasis	phase 3 completed	[117]
Fosmidomycin	Urinary-tract infections	Malaria	phase 2 completed	[117]
Harmine	Cancer	Malaria	preclinical	[148]
Miltefosine	Cancer	Visceral and cutaneous leishmaniasis	phase 2 completed	[117,149]
Paromomycin	Antiamebic	Visceral leishmaniasis	phase 4 completed	[117]
Pentamidine	Pneumonia (*Pneumocystis carinii*)	Trypanosomiasis and antimony-resistant leishmaniasis	phase 2 completed	[117]
Auranofin	Rheumatoid Arthritis	Amebiasis	Clinical use	[150]
**Anti-parasitic**				
Closantel	Antihelminthic	Onchocerciasis	preclinical	[148]
**Anti-prion disease**				
Quinacrine	Malaria	Creutzfeldt-Jakob Disease	phase 2 completed	[117]
***Others***				
Arsenic	Tuberculosis and syphilis	Acute promyelocytic leukemia	phase 2 completed, phase 3 active	[117]
Digoxin	Congestive heart failure and arrhythmia	Cancer	phase 1 completed, recruiting subjects for phase 2	[148]
Fumagillin	Antiamebic	Cancer (angiogenesis inhibitor)	preclinical	[117]
Gemcitabine	Antiviral	Cancer	phase 2 active	[149]
Itraconazole	Antifungal	Angiogenesis inhibitor	phase 2 active	[148]
Glefenine	Analgesic	Chemotherapeutics for tumor resistance	preclinical	[148]
Mycophenolic acid	Immunosuppresive drug	Angiogenesis inhibitor	phase 2 active	[148]
Nitroxoline	Urinary-tract infections	Angiogenesis inhibitor	preclinical	[148]
Retinoic acid	Acne	Acute promyelocytic leukemia	phase 2 completed	[117]
Riluzole	Amyotrophic lateral sclerosis	Melanoma and other cancers	phase 2 active	[148]
Thalidomide	Sedative / antiemetic	Cancer (angiogenesis inhibitor), erythema nodosum leprosum	phase 2 active	[117,149]
Bupropion	Antidepressant	Smoking cessation	phase 3 completed	[149]
Ceftriaxone	β-lactamase antibiotic	Amyotrophic lateral sclerosis	phase 3 completed	[117]
Dapoxetine	Antidepressant, analgesic	Premature ejaculation	phase 3 completed	[149]
Doxepin	Antidepressant	Insomnia, antipruritic	phase 2 completed	[149]
Duloxetine	Antidepressant	Urinary incontinence (stress-related)	phase 3 completed	[149]
Finasteride	Benign prostatic hyperplasia	Male baldness	phase 3 completed	[149]
Fluoxetine	Antidepressant	Premenstrual dysphoria	phase 4 completed	[149]
Hydroxychloroquine	Antiparasitic	Arthritis, systemic lupus erythematosus	recruiting subjects for phase 3	[149]
Milnacipran	Antidepressant	Fibromyalgia	phase 3 completed	[149]
Minocycline	Antibiotic	Amyotrophic lateral sclerosis	phase 3 completed	[117]
Mycophenolate mofetil	Immunosuppresive drug (transplant rejection)	Renal symptoms of systemic lupus erythematosus	phase 3 completed	[149]
Naltrexone	Opioid addiction	Alcohol withdrawal	phase 4 completed	[149]
Pioglitazone	Type-II diabetes	Nonalcoholic steatohepatitis	phase 2 completed	[149]
Raloxifene	Breast cancer	Osteoporosis	phase 3 completed	[149]
Ropinirole	Antihypertensive	Parkinson's disease, restless legs syndrome	phase 3 completed	[149]

### 3.2. RNAi Screens for Human Fungal Pathogens

*Cryptococcus neoformans* and *Candida albicans* are important human fungal pathogens responsible for extensive infections in the immune compromised or immune suppressed. RNAi analysis of genes essential for *C. albicans* phagocytosis revealed that of the ~7,200 *Drosophila* genes tested, 184 genes were required for efficient fungal phagocytosis of which macroglobulin complement related (Mcr) binds to *C. albicans* and promotes its uptake by phagocytes [83]. A similar analysis of host genes involved in replication of *C. neoformans* identified 57 genes that regulate phagocytosis, intracellular trafficking, replication, cell-to-cell spread, and escape. Major among these factors were autophagy associated factors Atg2, 5, 9, and PI3K59F that were recruited to the vicinity of infected vacuoles and involved in parasite trafficking [84].

### 3.3. RNAi Screens for Human Protozoan Pathogens

Kinetoplastid and Apicomplexan protozoan parasites are the third most important cause of human diseases worldwide, and invade multiple target cell types to complete their life cycle. Multiple virulence factors and host factors are involved in this process, and it is essential to identify the host factors to determine disease intervention strategies. RNAi screens targeting 727 human kinases identified five (MET, PKCζ (PKCzeta), PRKWNK1, SGK2, and STK35) genes essential for *Plasmodium falciparum* replication [85], and using RNAi, the host scavenger receptor SR-B1 has been shown to regulate *Plasmodium* infection [86]. Similarly, RNAi mediated knockdown of four *Anopheles gambiae* (the main vector for *Plasmodium transmission*) genes identified oxr1, argK, and prs1 [87]. In the case of *Trypanosoma cruzi,* a recent RNAi screen [88] targeting ~21,000 human genes identified 162 genes required for parasite growth and replication. Previous studies showed that RNAi knockdown of laminin γ-1 [89], thrombospondin-1 [90], and cytokeratin 18 [91] silenced *T. cruzi* infection or replication. A significant number of current drugs have been found to have significant anti-protozoal activity and are listed in Table 3.

### 3.4. RNAi Screens for Human Viral Infections

The RNAi pathway evolved to respond to intracellular infections. Although siRNAs have been shown to silence virus replication *in vitro* by targeting critical viral genes, a major concern for siRNA-based therapeutics is delivery of siRNAs, their pharmacokinetics, and the emergence of escape mutant viruses [92,93]. Identification of host target genes that are essential for viral replication but not temporally for the host is a promising area for development of novel anti-viral therapeutics. Consequently, a large number of studies with both DNA and RNA viruses have identified host genes/pathways that are crucial for viral infection/replication. It is important to note that screening methodologies differ even among the same viruses studied, thus while these screens have generated a substantial list of pro- and anti-viral genes, overlaps are minimal between screens. A major benefit of these RNAi screens has been to shortlist pathways that can be targeted for novel drug targeting, drug repurposing, and discovery. The majority of RNAi screens for determining host factors essential for viral replication have focused on HIV and influenza which are responsible for global epidemics, but recent studies have focused on other important human viruses including measles, herpes viruses, West Nile virus (WNV), hepatitis viruses, human papilloma virus (HPV), dengue virus (DENV), vaccinia virus, and others which are discussed below. 

It was first reported that RNAi silencing of the human transcription elongation factor SPT5 modulated the replication of Human Immunodeficiency virus (HIV) [94]. Human splicing factor DBR1 knockdown was consequently shown to modulate HIV replication [95]. A subsequent screen targeting 5,000 human genes identified known (TSG101, furin, CXCR4) and novel (Pak1 and 3) genes important in HIV replication [96], and a related study [97] extended these findings targeting 21,121 human genes and identified 273 genes that decreased viral replication. In addition to 36 genes previously implicated in HIV pathogenesis, the study also discovered novel pathways such as karyopherin-mediated import of HIV genome, autophagy, and retrograde vesicular transport were discovered and validated. A parallel study [98], identified 311 host factors that are required for HIV replication, and although this study showed minimal overlap [97], it did identify conserved pathways and six genes (AKT1, PRKAA1, CD97, NEIL3, BMP2K, and SERPINB6) that were essential for HIV replication. A short hairpin RNAi screen was also performed to identify kinases and phosphatases important for HIV replication, and in this study 14 novel genes involved in MAPK, JNK, and ERK pathways, vesicular transport, and DNA repair were identified [99]. A subsequent study [100], generated shRNA overexpressing cells lines against 30 cellular factors implicated at different stages of HIV life cycle, and observed a reduced susceptibility of 50% of these cell lines to infection. Cell lines expressing shRNAs against ALIX, ATG16, and TRBP were resistant to HIV infection for up to 2 months showing that knockdown of cellular factors could inhibit infection and replication. Since a major component of HIV disease depends on the integration of the viral genome into the host chromosomes, one study investigated if RNAi knockdown of DNA repair pathway genes would modulate HIV replication and subsequently identified the short patch base excision repair (BER) pathway genes as important for HIV replication [101]. RNAi screens targeting 19,121 human transcripts also identified a critical role for the PAF1 complex in HIV down-regulation, where siRNA knockdown of the PAF1 family of proteins enhanced HIV-1 reverse transcription and integration of provirus while over expression of PAF1 made cells recalcitrant to HIV-1 and 2 infections [102]. Recently it was shown that RNAi-mediated stable knockdown of five autophagy factors in T cell lines resulted in inhibition of HIV replication without affecting cellular viability [103].

Several genome-wide RNAi screens have been performed for influenza using a variety of approaches. Greater than 100 genes were identified using a siGenome library covering 90% of the *Drosophila* genome that modulated influenza virus replication and ATPV601, COX6A1, and NXF1 validated in human HEK293 cells as critical for influenza virus replication [104]. In a related study [105], >120 host genes were identified that modulated influenza replication and Interferon-inducible transmembrane proteins IFITM1, 2, and 3 were shown to be important in restricting influenza replication, a feature which was also conserved for WNV and DENV infections. A different approach was undertaken using a yeast two hybrid screen that identified human proteins interacting with influenza viral proteins [106]. Salient features of this interaction network revealed an extremely interconnected network. This network of cellular proteins was found to be significantly deregulated upon viral infection, and 616 genes were shown to affect either viral replication and/or modulate IFN-β levels without affecting cellular viability. It has also been reported that 287 human host genes regulate influenza A virus replication [107], and validation of these genes revealed 168 genes regulating the replication of both an endemic and a pandemic swine origin strain. In this study, the SON family of DNA binding proteins was shown to be involved in influenza virus trafficking to late endosomes. Since influenza replication and transcription occurs in the nucleus, another group investigated the interactions between influenza viral polymerase complex (PB1, PB2, and PA) and nuclear proteins during both a H1N1 and H5N1 infection. The study showed that RNAi of multiple RNA binding proteins (DDX17, DDX5, NPM1, hsRNPM), stress related (PARP1, DDB1, and Ku70/86), and intracellular transport proteins significantly reduced viral polymerase activity and was strain dependent. RNAi studies identified 295 genes involved in early stage virus replication of which 219 were validated to be important for wild type viral replication [108]. The study further validated the role of 23 genes, including vacuolar ATPAses, COPI proteins, fibroblast growth factor receptor (FGFR) proteins, and glycogen synthase kinase 3 (GSK3) in viral entry, and 10 genes including nuclear import components, proteases, calcium/calmodulin dependent protein kinase II beta (CamKIIβ) in post entry events. Small molecule inhibition of several host factors such as vATPAse and CAMK2B were shown to modulate viral replication. Recently, a protease RNAi screen [109] demonstrated that ADAMTS7, CPE, DPP3, MST1, and PRSS12 modulate influenza virus replication and are regulated by microRNAs during infection.

A RNAi screen for Hepatitis C virus (HCV) that targeted 140 cellular membrane trafficking genes identified 16 host cofactors that are important for HCV infection [110]. A recent study compared human genes that regulate negative-strand RNA virus replication and identified 72 host genes that regulate vesicular stomatitis virus (VSV), lymphocytic choriomeningitis (LCMV), and human parainfluenza virus type 3 [111]. RNAi screens for emerging infectious agents such as Ebola have identified a number of proteins such as the Phosphatidyl-3 kinase and Calcium/Calmodulin kinase as host genes important for viral replication [112]. Pox virus infection was shown to be regulated by seven genes including three subunits of the protein kinase, AMP-activated, alpha 1 catalytic subunit (PRKAA-1/AMPK) as well as Cullin-3, and macro-pinocytosis of vaccinia virus was shown to depend on AMPK expression [113,114]. RNAi knockdown of RNAi components has been recently shown to be important for DENV replication [115], and recently 96 cellular proteins including bromodomain protein Brd4, demethylase JARID1C/SMCX, and components of the histone acetyl transferase complex were shown to interact with the human papillomavirus long control region promoter, and together with the HPV E2 protein contribute to viral E6 and E7 oncogene expression [50]. Similarly, an RNAi screen identified 117 human genes that modulate replication and infection of coxsackievirus B (CVB) and poliovirus (PV) of which 17 genes were anti-enteroviral while the remaining genes were pro-viral [51]. 

Human pathogens have evolved multiple mechanisms to overcome host detection and clearance of infection and RNAi screens help us to understand the mechanisms employed toward this. Table 2 provides a brief summary of the results of RNAi screens from various human pathogens to date. 

## 4. Combining Strategies to Target Host Genes

While RNAi and related approaches help identify cellular genes and networks in response to infection, stress, or stimuli, it is important to note that these are snapshots of the biological phenomena and do not necessarily reflect the dynamic nature of biological networks. A hallmark of genome-wide approaches is the overwhelming amount of information generated which must be analyzed and validated. As has been shown across variety of systems and models, this endeavor is not trivial as hits from primary screen must be validated using alternate approaches that phenocopy the endpoints evaluated in the primary screen. Alternative approaches to facilitate validation have utilized imaging-based high-content screening, shRNA screenings and/or deconvulation of siRNAs pools used in the primary screen. It is commonly observed that only 10-20% of primary hits make it through validation (Table 1 in [28]). A variety of bioinformatics tools [127,128] have facilitated this process, but cannot replace the need for human analysis and careful data interpretation. Off-targeting by siRNAs can be due to use of non-physiological concentrations of siRNAs, transfection reagents, and other aspects, and these needs to be filtered from the plethora of information to identify real targets. Complicating these interpretations is the incompletely understood contribution of siRNA effects on the endogenous microRNA pathways in cells. miRNAs contain a “seed region” (nt 2-8) at their 5´-end that binds to a complementary sequence (perfectly or imperfectly) in the 3’-UTR of a gene transcript causing transcript decay or blocked translation. Since siRNA sequences can contain miRNA seed sequences, there is a growing concern that off-target effects of siRNAs may be mediated by deregulating the activity and target repertoire of native miRNA populations, and these need to be teased out. Indeed, it was recently shown that proteases crucial for influenza virus replication [109] are also regulated by microRNAs during influenza infection adding an additional layer of complexity to the picture. This suggests that data from RNAi screens should be complemented with parallel studies of miRNA knockdown/upregulation to dissect out siRNA *vs.* miRNA regulated pathways during infection. Moreover since mRNA and miRNA expression profiles are cell type specific; these networks need to be analyzed in the context of the model system under investigation. One way that could facilitate this is the use of pathway analysis using existing models such as Ingenuity Pathway analysis (IPA) or other open source tools. Pathway analysis tools allow hits to be mapped to existing metabolic and other pathways, identify, and statistically score important nodes in the network and can aid in separating the needles from the haystack by analyzing existing literature databases for the hits. Many pathway tools are available [129,130,131] and the choice of one over another is a decision based on functionality, features, and cost that need to be determined by the researchers. Thus, an end-goal in undertaking an RNAi screen, whether genome-wide or targeted is to identify genes important for the biological question to be addressed, and in many cases, utilize the research findings to identify existing or novel drug candidates. The experimental design and assay methodologies described above combined with data analysis tools available can accelerate this process.

## 5. Rescuing and Repurposing Drugs

Development of new therapeutics drugs is a time consuming, expensive process associated with frequent failure. The average time line from drug development to the bedside takes ~15 years and costs >$1 billion with >95% drugs failing [117,132,133]. Thus, repurposing older and existing drugs, also known as drug rescuing, repositioning, or reprofiling, is an option that would allow for more rapid drug availability. Here, existing drugs with known safety profiles which have been successfully used for treatment of unrelated diseases, or failed late stages of clinical trials are evaluated for their efficacy against unrelated diseases [117,134]. Drug repurposing idea stem from promiscuous nature of drugs, in which a particular drug is able to target multiple molecules or pathways, and that each cellular molecule can take part in multiple biological pathways contributing to different diseases. Drug repurposing allows new treatments to be available in approximately two years while significantly reducing development costs by 40%.

One notable example of successful drug repurposing is the development of the first anti-retroviral drug azidothymidine (AZT), approved by the FDA only two years following demonstration of its anti-retroviral activity *in vitro*. AZT was initially developed for cancer treatment in the 1960s, but was subsequently withdrawn due to its lack of efficacy. Through a collaboration between the National Cancer Institute (NCI), the Burroughs-Wellcome Company (now GlaxoSmithKline), and Duke University, AZT was found to be effective against HIV and in 1987 was the first FDA-approved drug for treatment of HIV/AIDS, which had no known treatment at the time [135,136]. Examples of other repurposed drugs currently in different stages of clinical trials are listed in Table 3.

Despite these success stories, drug repurposing efforts are hampered by limited compounds made available by pharmaceutical companies for academic researches. To ease this problem and to promote a more rapid development of new therapeutics, the NIH’s National Center for Advancing Translational Sciences (NCATS) recently launched an initiative in “Discovering New Therapeutic Uses for Existing Molecules” in May 2012 (http://www.ncats.nih.gov/research/reengineering/rescue-repurpose/rescue-repurpose.html, [137]). This initiative provides academic researchers access to a library of compounds available for repurposing from eight major pharmaceutical companies: Abbott, AstraZeneca, Bristol-Myers Squibb Company, Eli Lilly and Company, GlaxoSmithKline, Janssen Pharmaceutical Research & Development, L.L.C., Pfizer, and Sanofi, and provides $20 million in research funds. Several other compound libraries are also available for drug repurposing research, such as the NIH’s Chemical Genomic Center (NCGC) Pharmaceutical Collection, the National Institute of Neurological Disorders and Stroke (NINDS) library, and the Johns Hopkins Clinical Compound Library (JHCCL) [117,138,139,140,141]. 

While several blockbuster drugs such as sildenafil (Viagra®) and minoxidil (Rogaine®) were results of serendipitous discoveries of their initial off-target effects [142,143,144], more targeted efforts toward drug repurposing should be performed rather than relying on serendipity alone. As described in the previous section, high-throughput RNA interference (RNAi) screens can be utilized for discovery of druggable genes, which potentially can be targeted for disease therapeutics. 

### 5.1. RNAi Screening to Identify Drug Targets and Drug Repurposing

As described earlier in this review, libraries containing collections of siRNAs or shRNAs have been developed and can be utilized to rapidly screen thousands of genes involved in particular disease-related biological processes. Specific libraries containing druggable host genes are also available and particularly valuable for discoveries of new drug targets. These libraries were compiled based on druggability properties of genes protein products, where folding of these proteins favors interaction with drug compounds, thereby increasing the chance of pharmacological inhibitors availability [30]. 

**Figure 2:**
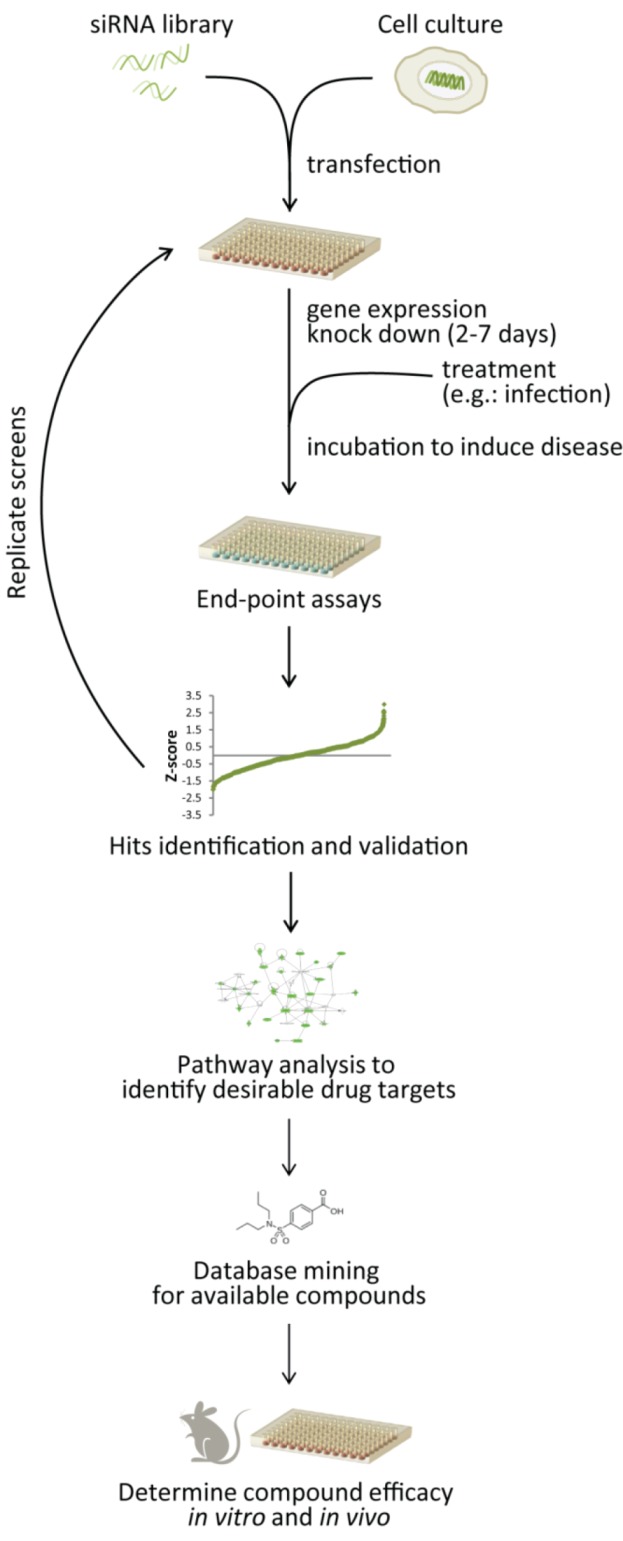
Strategies for siRNA screening process to identify new therapeutic target toward drug repurposing. Following primary and secondary screens, potential gene targets are further analyzed *in silico* using pathway analyses and database mining to determine desirable drug targets and available compounds. Efficacy and safety of these compounds can then be assessed *in vitro* and *in vivo* before clinical trials can be justified.

A general workflow of siRNA screening towards repurposing available compounds is shown in Figure 2. Typically, the screening process begins with transfection of an appropriate cell culture system with a siRNA library using conditions pre-optimized for the particular cell type followed by 42-72 hour incubation to allow for maximal reductions of gene expression. Subsequently, cells can be induced or treated as required in the experimental design or disease model, *i.e.* infection with pathogen of interest, or induction of a particular disease such as cancer or injury. Cells are then evaluated at desired time points using pre-determined endpoint assays to yield hits. List of gene hits, containing genes that demonstrated through the siRNA screen to modulate disease, are then compiled. These genes are then validated using other relevant cell culture models (different cell types, viruses, induction, *etc.*), different end-point assays, by deconvoluting the siRNA pool to individual siRNAs, and/or by using different siRNA targeting a different seed site on the same gene. These genes can be potentially targeted for therapeutic disease intervention strategy.

Following secondary screens and validations, bioinformatics can then be employed to analyze potential gene targets, such as utilizing gene ontology (GO), pathway mapping, and interaction databases (GeneGo MetaCore™, IPA, Toppcluster) to further understand host response networks involve in particular disease [130,151]. Ideal therapeutic targets, such as an upstream regulator or a node in the pathway can be determined from these *in silico* analyses. Once potential targets have been narrowed, database mining can be employed to find available small molecules or drugs targeting these genes. Multiple databases containing currently available drugs and their gene targets are publically available, such as: the PROMISCUOUS database, ChemSpider, and DrugBank as summarized in Table 4. Other drug databases and references such as PubChem (http://pubchem.ncbi.nlm.nih.gov), ChEMBL (https://www.ebi.ac.uk/chembl), and the Clinician’s Pocket Drug Reference (Scut manual) can also be used for this purpose. Candidate drugs can then be tested *in vitro* and *in vivo* for their efficacy as disease therapeutics, before initiating clinical trials. 

**Table 4:** Examples of comprehensive online databases containing available drugs and their gene targets to aid drug repurposing efforts.

Name	Website address	Contents	Ref.
PROMISCUOUS	http://bioinformatics.charite.de/promiscuous	A database containing 25,000 annotated withdrawn or experimental drugs searchable by name, target, or pathway.	[141]
ChemSpider	http://www.chemspider.com	Free drug database containing 28 million structures searchable by calculated properties, structures, or drug ligands.	[152]
DrugBank	http://www.drugbank.ca	A comprehensive database hosted by the University of Alberta that contains 4,800 drugs including FDA-approved small drugs, natural agents, and experimental drugs and their sequence, structure, and target pathway.	[140]

## 6. Case Studies for RNAi Screening towards Drug Repurposing

A majority of repurposed compounds listed in Table 3 were identified through small molecules screens using available libraries such as the NCGC, NINDS, and JHCCL libraries noted. In addition to these repurposed compounds, several studies have successfully utilized RNAi screens to identify disease-associated cellular processes, and have demonstrated the ability of available compounds to target cellular factors to improve disease *in vitro* and/or *in vivo*. Several of these studies are described in more detailed below; first two are examples related to viral therapeutics, while the last example is related to cancer therapeutics.

### 6.1. Identification of OAT3 as Pro-influenza A Host Factor and Repurposing of OAT3 Inhibitor Probenecid

Influenza virus infection is a significant public health concern with a high pandemic potential, resulting in over 250,000 hospitalizations and up to 49,000 deaths occur annually in the United States alone [153]. Several anti-influenza drugs are available, such as the neuraminidase (NA) inhibitors zanamivir (Relenza®) and oseltamivir (Tamiflu®), and the M2 ion channel inhibitors amantadine and rimantidine [154,155,156]. These antiviral drugs target viral components which rapidly drive selective pressure for drug resistance. Unfortunately, only a limited number of new anti-influenza drugs are currently in development while drug resistance is growing in circulating and pandemic influenza virus strains [157], thus there is a need to develop new classes of anti-influenza chemotherapeutics. Several recent studies have utilized RNAi screens to identify host cellular factors involved in influenza A virus replication [36,104,105,106,107,108,109,158,159]. Targeting these pro-viral host factors can serve as therapeutic strategies for treatment of influenza virus infection. One pro-viral factors identified through a siRNA screen using the SMARTpool siGenome drug target library containing 4,795 human druggable genes was the organic anion transporter-3 (OAT3) [159]. The prototypical OAT inhibitor probenecid is currently widely prescribed as treatment for gout and other hyperuricemic disorders [160,161]. This study demonstrated probenecid had efficacy to reduce influenza virus replication *in vitro* and *in vivo* when given prophylactically or therapeutically. Probenecid also effectively reduces influenza virus-associated mortality and morbidity *in vivo* [159]. Interestingly, probenecid was previously shown to sustain plasma levels of oseltamivir active metabolite and its co-administration with oseltamivir was suggested [162,163,164]. This study also confirmed the efficacy of oseltamivir-probenecid combinational therapies *in vivo*, which can now be attributed to both probenecid’s direct anti-influenza property and its role to sustain plasma oseltamivir level. This study exemplifies how RNAi screening can be utilized to identify a new drug targets for anti-influenza A therapy, and that widely prescribed drugs like probenecid can be potentially repurposed as a new anti-influenza A therapeutic. 

### 6.2. Identification of Host Proteins in Phosphatidylinositol-3-Kinase and Calcium/Calmodulin Kinase-Related Pathways Important for Zaire Ebola Virus Entry and Its Inhibition by Known Inhibitors

Ebola virus is an etiologic agent of Ebola hemorrhagic fever (EHF) which is characterized by the sudden onset of fever, malaise, muscle pain, headache, and sore throat, followed by vomiting, diarrhea, rash, kidney and liver failure, internal and external bleeding, and eventually death is many cases [165]. Zaire Ebola virus (ZEBOV), one of the five species within the Ebola virus genus, has the highest average case-fatality rate of 83% since its first discovery in 1976 in a small village located along the Ebola river in northern Democratic Republic of Congo (previously known as Zaire). Ebola virus is transmitted through close contact with organs, blood, or other bodily fluids of infected people and quarantine has been the most effective method to contain Ebola virus outbreak. There is no available vaccine or effective therapeutics against Ebola virus infection, although ZEBOV-infected patients are often treated with broad-spectrum antiviral ribavirin despite its lack of reported efficacy [165,166]. Similar to the influenza A virus study described above, a screen using siRNA library of host druggable genome containing 720 kinase and other phosphorylase genes was performed to identify host factors involved in Zaire Ebola virus (ZEBOV) [112]. This study utilized a pseudotyped lentivirus expressing ZEBOV glycoproteins and luciferase (ZEBOV-Luc), which allowed for high-throughput detection of infection. This study identified that host proteins in the phosphatidylinositol-3-kinase (PI3K) and calcium/calmodulin kinase (CAMK) pathways play significant roles for ZEBOV pseudotyped lentivirus entry. Furthermore, chemical inhibitors of PI3K and CAMK2 proteins LY294002 and KN-93, respectively, were demonstrated to block entry of (ZEBOV-Luc) and ZEBOV pseudotyped vesicular-stomatitis virus, and importantly the wild-type ZEBOV under BSL4 condition. Thus, LY294002 and KN-93, both of which are currently developed as cancer therapeutics, can also be potentially repurposed as anti-Ebola antivirals. Interestingly, in two separate siRNA screening studies, CAMK2 has also been identified to have an important role during influenza A virus infection, and CAMK2 inhibitor KN-93 shown to limit influenza virus infection [108]. Collectively, these studies indicate CAMK2 inhibitor KN-93 potential as antiviral therapeutic against multiple RNA virus infections.

### 6.3. siRNA Screen Reveals Chemical Inhibitor to Prevent MYC-Driven Oncogenesis

Deregulation and constitutive expression of cellular MYC (c-MYC) oncogene is associated with poor prognosis of many human cancers, including a higher rate of metastasis, recurrence, and mortality [167]. However, targeting c-MYC itself is not a plausible strategy for therapy due to its lack of druggable property and its essential role for normal cellular proliferation [30,168]. A recent study utilized a siRNA screen to identify cellular factors essential for proliferation and oncogenesis associated with c-MYC overexpression [169]. This study targeted ~3,300 druggable genes and 200 microRNAs using primary human foreskin fibroblasts (HFFs) overexpressing c-MYC through retroviral transduction in 384-well HTS format. Reduction of viability of c-MYC-overexpressing cells following gene silencing was assessed to identify cellular factors associated with MYC-driven cancer, termed MYC-synthetic lethal genes. Cellular factors associated with normal cellular proliferation, assessed also by siRNA screen of HFF cells in the absence of c-MYC overexpression, were then deducted from the gene list. This study identified 102 MYC-synthetic lethal hits, which include genes with known association with MYC and MYC-related genes, and other genes associated with diverse cellular processes such as in basic transcriptional machinery, ribosomal biogenesis, chromatin modification, metabolism, DNA repair, apoptosis, and mitotic control as analyzed by pathway analysis. Several gene hits, including the casein kinase 1 epsilon (CSNK1ε) gene were validated using gene-specific shRNA. Additionally, this study demonstrated that small molecule inhibitor of CSNK1e enzymatic activity, IC261, can reduce relative viability of c-MYC overexpressing HFF cells, in addition to MYC-overexpressing Tet21N cells and MYCN-amplifying IMR-32 neuroblastoma cells. Importantly, IC261 was observed to reduce tumor growth *in vivo* using IMR-32 neuroblastoma xenograft mouse model. Thus, this study provides yet another example of how RNAi screens can be utilized to identify disease-associated cellular factors and how these findings may lead to identification of new drug targets and repurposing of available drugs. 

## 7. Going Forward with RNAi Screens and Drug Repurposing

While the approaches described in this review identify RNAi-based screening as an approach to identify novel genes and drug repurposing candidates, a careful approach in designing, planning and execution of the screen is absolutely essential. Time-points and endpoint assays need to be extensively optimized and validated to ensure that biological noise does not mask significant endpoint observations, nor are false positives identified. While model systems provide great flexibility in the screening, they are typically removed from physiological conditions and hence data needs to be interpreted with caution. Rigorous validation using independent secondary assays and additional time points can help narrow down real candidates. It should also be noted that while repurposing offers several advantages over novel discovery, it is not always possible to identify relevant small molecules following an RNAi screen. Repurposed drugs may not exhibit significant activity in the model system used or may have extensive toxicity precluding their use in the clinical setting. Currently, alternative low cost and rapid approaches to identifying relevant host cell pathways are not generally available, thus drug repurposing remains an efficient approach towards developing alternative therapeutics for existing diseases. 

## 8. Conclusions

A growing body of literature has demonstrated the benefit of performing genome-wide siRNA screens to understand biological processes. A variety of reagents for introducing siRNA, miRNAs, and other silencing constructs such as shRNAs have been commercially and academically developed to target genes in traditionally easier as well as harder cell types to transfect. Screening approaches are generally validated using independent assays and novel technologies to understand the complexity inherent to gene knock-down. Computational approaches and high throughput screening bioinformatics have helped in analyzing the enormous amount of data generated from these screens to shortlist potential gene/pathway targets. One important outcome of the RNAi screening approach has been the identification of genes that can be targeted by existing drugs or small molecules leading to drug repurposing. Numerous studies demonstrated that RNAi screening can be utilized as a fast, data driven, yet low cost approach to bringing new treatments to the clinics. Furthermore, RNAi screening has significantly contributed to our understanding of biological systems, and how they respond to environmental and other stimuli. Addressing challenges such as small molecule specificity and delivery methods will significantly bolster development of novel therapeutics.
